# Relatives Education And Coping Toolkit - REACT. Study protocol of a randomised controlled trial to assess the feasibility and effectiveness of a supported self management package for relatives of people with recent onset psychosis

**DOI:** 10.1186/1471-244X-11-100

**Published:** 2011-06-16

**Authors:** Fiona Lobban, David Glentworth, Laura Wainwright, Vanessa Pinfold, Lesley Chapman, Warren Larkin, Graham Dunn, Adam Postlethwaite, Gillian Haddock

**Affiliations:** 1 Spectrum Centre for Mental Health Research, School of Health and Medicine, Lancaster University, Lancaster, LA1 4YT, UK; 2 Bolton EIS, Paragon Business Park, Chorley New Road, Horwich, BL6 6HG, UK; 3 Rethink, 15th floor, 89 Albert Embankment, London, SE1 7TP, UK; 4 Early Intervention Service, Lancashire Care NHS Foundation Trust, Daisyfield Mill, Appleby Street, Blackburn, BB1 3BL, UK; 5 Health Sciences Research Group, Jean McFarlane Building, Oxford Road, Manchester, M13 9PL, UK; 6 Division of Clinical Psychology, School of Psychological Sciences, S29 Zochonis Building, University of Manchester, Brunswick Street, Manchester, M13 9PL, UK

## Abstract

**Background:**

Mental health problems commonly begin in adolescence when the majority of people are living with family. This can be a frightening time for relatives who often have little knowledge of what is happening or how to manage it. The UK National Health Service has a commitment to support relatives in order to reduce their distress, but research studies have shown that this can lead to a better outcome for service users as well. Unfortunately, many relatives do not get the kind of support they need. We aim to evaluate the feasibility, acceptability and effectiveness of providing and supporting a Relatives' Education and Coping Toolkit (REACT) for relatives of people with recent onset psychosis.

**Methods:**

The study is a randomised control trial. Trial Registration for Current Controlled Trials ISRCTN69299093. Relatives of people receiving treatment from the Early Intervention Service for psychosis are randomly allocated to receive either Treatment As Usual (TAU) or TAU plus the REACT intervention. The main aims of the study are to: (i) determine the acceptability of a supported self-management intervention; (ii) determine preference for type of support; (iii) assess the feasibility of the design; (iv) identify the barriers and solutions to offering support for self-management approaches within the NHS; (v) estimate the likely effect size of the impact of the intervention on outcome for relatives; (vi) gain detailed feedback about the barriers and solutions to using a self-management approach; (vii) describe the way in which the intervention is used. Outcomes will be assessed from baseline and at 6 month follow-up.

**Discussion:**

The intervention is compared to current treatment in a sample of participants highly representative of relatives in routine early intervention services across the UK. The intervention is protocolised, offered within routine practice by existing staff and extensive process data is being collected. Randomisation is independent; all assessments are made by blind raters. The limitations of the study are the lack of control over how the intervention is delivered, the short follow-up period, and the lack of assessment of service user outcomes. Despite these, the findings will inform future effectiveness trials and contribute to the growing evidence base for supported self-mangement interventions in mental health.

## Background

Psychosis affects approximately 1% of people and is the third most disabling condition worldwide [1]. First episode commonly occurs in adolescence at which time it is estimated that 60-70% will be living with their family [2]. The government recognises the very important role of relatives and is committed to providing them with appropriate support via NHS services [3]. Family interventions (FIs) are effective in improving outcome for people with psychosis and their relatives. As an adjunct to pharmacotherapy, FIs reduce relapse and hospitalisation rates [4,5]. FIs generally focus on cognitive and behavioural techniques to modify appraisals that relatives hold about the behaviour of the person with psychosis and develop coping strategies. Research is limited in its focus on people with more chronic mental health difficulties, and lack of attention to outcomes for relatives. However, interventions that are well integrated into Early Intervention Services (EIS) show reductions in relatives' distress [6], and appraisals held by relatives at first episode of psychosis are significant predictors of important determinants of outcome [7].

Significant barriers still exist to the dissemination of effective interventions through NHS EIS. These include clinicians with high caseloads and lack of confidence and training in working with relatives [8]. As a result, relatives report significant negative impact on many areas of their life, and the risk of distress is even higher at first episode than at later stages [9].

There is a clear need for an intervention that can be widely available to relatives, is easy to use, phase specific, recovery focussed, does not require extensive clinical resources, targets key appraisals and coping strategies and empowers relatives. Self-management interventions that have the flexibility to be used alongside other work and family commitments and augment other forms of support are ideally suited to meet the needs of relatives. Self-management refers to health technologies (written/audio/video/computer/internet) to assist users to manage a particular health problem, with little or no professional input. They can be used as stand-alone interventions or as an adjunct to other forms of intervention. This is a rapidly growing area and a recent meta-analysis of studies evaluating such approaches for depression shows promising results [10]. Greater effectiveness is associated with using a 'guided' model with low-level contact with a professional/paraprofessional, and a CBT (Cognitive Behaviour Therapy) rather than educational model [10]. Self-management approaches can increase dissemination of evidence based interventions to large numbers of people, and foster empowerment. Although much self-help literature has been written for a wide range of mental health problems, there is little development in the area of psychosis and few high quality evaluations from which conclusions can be drawn regarding effectiveness [11]. We are not aware of any studies which have systematically evaluated the use of a supported self-management approach for relatives of people with psychosis.

This paper describes the rationale and protocol for a randomised controlled trial in which relatives of people at first episode psychosis in EIS receive current treatment or current treatment plus the REACT supported self-management toolkit. The main aims of the study are (i) to determine the acceptability of a supported self-management intervention and outcome measures to relatives of people with recent onset psychosis; (ii) to determine preference for type of support (email/telephone); (iii) to assess the feasibility of the design as measured by rates of recruitment, retention, attendance and direct feedback from participants; (iv) to identify the barriers and solutions to offering support for self-management approaches for relatives of people with psychosis in the NHS; (v) to estimate the likely effect size of the impact of the intervention on outcome for relatives; (vi) to gain detailed feedback from relatives about the barriers and solutions to using a self-management approach; (vii) to describe the way in which the intervention is used. This is not intended as a 'definitive' randomised controlled trial and will not assess the impact on patient outcomes or cost effectiveness. Further funding will be sought to address these issues if this study supports the feasibility.

## Methods

This trial is conducted by a multidisciplinary team of researchers, clinicians, statistician and relatives based across academic institutions and NHS Trusts in the North West of England. The research team are responsible for the content of the intervention and have worked with a design company to produce the toolkit and website. The trial is supported by an independent Trial Steering Committee.

### Design

#### Randomisation, Treatment Allocation and Blinding

**Figure 1:**
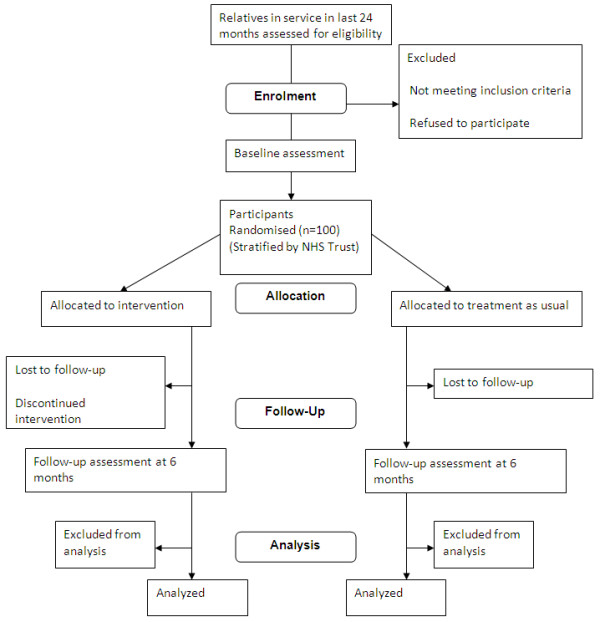
**Consort diagram showing progression of participants through the trial**.

This is a stratified randomised controlled trial in which participants are allocated to receive either Treatment As Usual (TAU) or TAU plus the REACT intervention. Randomisation is done using permuted blocks within Trust with block sizes varying randomly, and is carried out by an independent Clinical Trials Unit at The Christie NHS Foundation Trust, Manchester. Assessments are carried out in face-to-face interviews at baseline and at 6 months follow-up. Participants are referred into the study by Care Coordinators or self-referral. Potential participants are contacted by a research assistant (RA1) who presents them with verbal and written information about the study. If they wish to take part, the participant is asked to give written consent. Following this, a baseline interview is conducted to assess eligibility and to complete all the measures. A second research assistant (RA2) then contacts the Clinical Trials Unit and provides information about which Trust the relative is in. They are given the trial allocation by telephone. RA2 contacts the relative by telephone and post to inform them which arm of the trial they have been allocated to. RA2 contacts the relevant support worker who will guide the relative through the intervention and instructs them to arrange the first appointment with the participant. RA2 also arranges the 6 month follow-up interview date. In order to ensure that outcomes are collected blind to the treatment allocation, a letter is sent prior to this interview reminding the participant about the importance of not letting RA1 know which group they were allocated to, and making sure that the REACT toolkit is not visible in the house. This is followed-up with a phone call the day before the interview to check the appointment time is convenient and to reiterate the need to maintain blindness. RA1 conducts all of the follow-up interviews and remains blind to allocation throughout the study. To ensure blindness, all communication between relatives and NHS staff is via RA2. The RAs are housed in separate offices and receive individual supervision. Any instances of unblinding will be recorded (Figure 1).

This study was reviewed and approved by the UK NHS Ethics Committee process (REC ref: 08/H1001/147).

#### Qualitative evaluation

Qualitative interviews will be used to help us understand how the intervention is used and experienced. Relatives participating in the intervention arm of trial will form the strategic sampling pool and will be invited to take part in lightly structured interviews to explore key domains around their use of and experience of the intervention, focusing on the barriers to using the intervention and potential solutions to overcome these. Recruitment will continue until data saturation is reached (estimate approx 10-15 relatives).

### Participants

#### Inclusion criteria

Three NHS Mental Health Trusts in the North West UK are taking part in this study. Each trust has an Early Intervention Service for Psychosis team which supports young adults who are experiencing symptoms of psychosis for a period of up to 3 years. Participants are relatives, partners or close friends of people currently receiving support from one of these teams. Additional criteria include: first contact with EIS within the last 24 months; sufficient understanding of written and spoken English to be able to use the intervention; aged over 18.

#### Recruitment and consent

Only relatives who already have direct contact with each EIS, and for whom the service has current contact details are invited to take part in the research. This includes relatives currently on the "family and friends group" lists, currently attending groups, and currently receiving face-to-face input from clinical staff and support workers. No relatives are contacted using details taken from patient medical notes without patient consent. No information is collected about the service user. Therefore, service users will not be required to consent into the study. However, to ensure service users are aware of the study and could invite any of their relatives not currently involved with the service, each service will write to all service users informing them about the study and send them a copy of the relatives' information sheet with a covering letter asking them to pass this to any relatives that they would be happy for us to invite and who they feel may be interested in taking part.

Care Coordinators will be informed about the study in a series of presentations given by the research team. They will be asked to discuss the study with relatives they are working with and to refer any relatives interested in taking part who consent to their details being given to the research team. A series of presentations will also be made to relatives at any service user and care events that happen in the Trusts during the recruitment phase. These will encourage relatives to refer themselves into the study if they wish to take part. A website will also be set up that enables confidential referrals to be made either by Care Coordinators or directly from relatives.

#### Sample Size

The main aim of the study is to assess the feasibility of the design and intervention. The sample required for this is not based on a formal power calculation but on a pragmatic decision balancing sufficient numbers to be able to identify likely barriers to carrying out a larger scale trial, and cost. We aim to recruit 100 participants which will also allow us to estimate the likely effect size of the intervention on a range of outcome measures.

### Outcome measures

Relatives will be assessed on a range of measures at both baseline and follow-up. All measures are conducted in a face-to-face interview.

1) The General Health Questionnaire (GHQ-28) [12] is a 28-item version of General Health Questionnaire derived by factor analysis from the full 60-item version. It is used to assess the psychological aspect of quality of life or in the detection of psychiatric distress. Participants indicate whether their current state differs from their usual state, thereby assessing recent changes in state as opposed to long-term traits or illnesses.

2) The Family Questionnaire [13] presents participants with a range of symptoms of mental illness that have the potential to pose a problem in family life. It assesses the extent to which each of these symptoms cause the relative concern and how well the relative is able to cope with their concerns.

3) The Relationship Quality Scale [14] asks service users and relatives to rate their perception of their relationship with one another on scales of supportiveness and criticism. This measure has been shown to predict outcome for people with severe depression [14] and schizophrenia [15].

4) The Brief Illness Perception Questionnaire [16] adapted from the original Illness Perception Questionnaire [17] measures the insight of participants into their relative's illness. Components include the participant's views on the consequences of the illness, comprehension of the illness, knowledge of prognosis, the extent to which they attribute blame, knowledge of potential causes and their own concern and emotional responses to the illness.

5) The Herth Hope Index [18] consists of 12 items designed to assess hope in adults in clinical settings.

6) The Experience of Caregiving Inventory [19] measures the experience of caring for a relative with a serious mental illness. The 66-items are divided into 10 sub-scales, 8 negative (difficult behaviours, negative symptoms, stigma, problems with services, effects on the family, the need to provide back-up, dependency, loss) and 2 positive (rewarding personal experiences, good aspects of the relationship with the patient).

7) The Carer Well-Being and Support Questionnaire [20] measures the experience of carers of people with severe mental health problems. It is designed to cover all aspects of the carer's experience including relationships, roles, financial concerns, physical/emotional health, stigma, worries about safety, their satisfaction with support offered and ease of obtaining information.

8) The Relatives' Satisfaction Questionnaire (adapted from CSQ-8 [21]) is used to assess satisfaction with services in both arms of the trial to test the hypothesis that the intervention will lead to an increased level of satisfaction in relatives.

9) The Treatment As Usual Checklist is a short questionnaire designed for this study to assess the amount and type of support that participants have received from the Early Intervention Service while in the Treatment as Usual arm of the trial.

### The Intervention

#### Development

**Figure 2:**
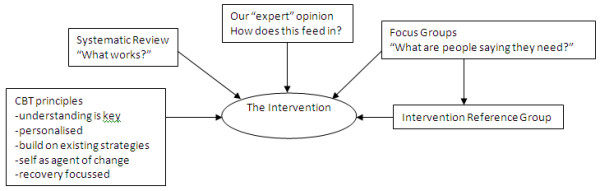
**Development of the REACT Intervention**.

During the first year of the study a CBT oriented, supported self-management package for relatives of people with a recent onset psychosis was developed and finalised. First, a systematic review of studies of psychological interventions for relatives of people with recent onset psychosis was conducted. This identified the key components of effective interventions and distinguished them from those of ineffective interventions. Second, relatives of people who have experienced psychosis were invited to take part in focus groups to allow in-depth analysis of relatives' views and experiences of a self-management approach. The questions asked, focussed around (i) their experiences of self-management approaches; (ii) preferred format (or "health technology"); (iii) what support they would like to receive; (iv) perceived barriers. Further details on the findings of this study are reported elsewhere [22]. Finally, participants from the focus groups were invited to be part of an Intervention Reference Group that was involved in an iterative process of feedback and development to produce the supported self-management package. An independent design company was used to style the package professionally and an independent publisher was used to produce the required number of intervention packs (Figure 2).

#### Features

The finalised package is modular in design, providing a range of sections or "tools" that relatives can draw on as needed. These modules have been designed to operate independently of each other and contain topics such as essential information about psychosis, ways to identify and challenge beliefs that may cause distress, ways to manage common difficulties faced by relatives, and coping strategies to aid recovery for both the relative and the person with psychosis. The package is also well referenced to guide people to existing support in related domains such as legal advice, advocacy, charities etc. Building on previous research in self-management for depression, the intervention also gives relatives the opportunity to incorporate their personal experience into the process, to situate this intervention within a context of previous experiences, build on existing self-management strategies to facilitate engagement, and highlight the self as the key agent of change [23].

The intervention has been designed to be used by relatives in their own homes and at their own convenience. As such, it has been produced as a hard copy format and as a website and participants are able to choose to use either or both of these formats. Both versions contain the same information and resources. Support is provided by an NHS support worker trained and supervised by the research team. The support worker will offer an initial face-to-face introductory session in which they guide the relative through the materials and how to use them. Following this, support in using the package will be offered via email or telephone depending on the relatives' preference.

### Analysis

#### Quantitative data

Analysis of outcomes will be carried out using simple descriptive statistics, tabulation and simple graphical display. Tests of statistical significance will be carried out using Student's t-test (quantitative outcomes) or Pearson chi-square (binary outcomes). Further analyses to allow for pre-randomisation (baseline) measures and to investigate the effects of missing outcomes will be carried out using analyses of covariance or logistic regression, respectively.

#### Qualitative data

Analysis will be carried out by a multidisciplinary team of psychologists, nurses, relatives and researchers. To ensure that the analysis is grounded in the data, rather than reflecting pre-existing ideas, analysis will occur in parallel with data collection so that aspects of the developing analysis can be tested in subsequent interviews. Categorisation and thematic analysis of the data will be developed by cycling between the analysis and transcripts and periodic 'testing' of the analysis by discussion amongst the entire team so as to meet accepted criteria for trustworthiness of the analysis. In addition, we will assess the validity of the final analysis by examining coherence and catalytic validity, which is essentially the utility of the analysis in identifying implications for clinical practice and research that can be tested.

## Discussion

The REACT toolkit has the potential to offer relatives the information and support they need during the crucial period of early psychosis, in a format which is highly accessible, free to access and can be used flexibly to meet their individual needs. It offers NHS Trusts the potential to meet government guidelines for supporting relatives, whilst overcoming many of the barriers associated with training and resources in offering face-to-face family interventions. The content of the intervention is based on already established effective interventions, but important questions need to be answered about whether these interventions can be offered in a supported self-management format. This trial will answer key questions of feasibility that need to be addressed before a large scale clinical and cost effectiveness evaluation of this approach. Specifically, the trial will provide extensive quantitative and qualitative data on the acceptability of the intervention for relatives, exactly how they use the intervention, and their preferences for how this should be delivered, including format of the toolkit and type and amount of support required to use it. Barriers and facilitators to using the toolkit and support will be identified from the perspective of the relatives, but also from the clinical staff attempting to deliver REACT. Important data for future research will include feasibility of recruitment, and retention to a large scale trial, and estimates of effect sizes on key outcome variables which can inform future power calculations.

This trial benefits from rigorous design in terms of independent randomisation, blind rated assessments, and a clearly protocolised complex intervention. In addition, the research is conducted within a "real world" setting, recruiting relatives from existing NHS services, delivering the intervention alongside current treatment, and offering support via existing clinical staff. This increases the external validity of the findings, but is also the source of some potential limitations. These include the variation that will undoubtedly exist in the way in which the intervention is supported, and variation within the current treatment offered as the control condition. Variation in both is likely to be present at an operational level between the Trusts taking part, but also for each participant, given their individual journeys through mental health services. Extensive process measures are being collected in an attempt to measure this variation with a view to understanding potential confounds in future effectiveness trials. Additional limitations include a follow-up period of 6 months only, and lack of service user outcomes. Both can be addressed in future trials but are beyond the scope and resources of a feasibility trial.

REACT is designed to provide information and strategies for relatives to build upon their existing coping strategies. It is not designed to replace face-to-face contact with Care Coordinators who provide valuable emotional support and detailed specific information about the service user that relatives are so keen to understand. Neither is this approach designed to replace intensive family therapy that should be offered to those families where difficulties arising from the psychosis have caused major breakdown in communication within the family, or severe psychological distress for the relatives. All relatives should have direct access to crisis services. Finally, many relatives derive benefit from receiving support from other relatives who have also experienced psychosis within their family. In its current form, this toolkit fails to offer this peer support that is available in many Early Intervention Services via family and friends support groups. Although REACT has been designed with user involvement to meet the needs of relatives, it is important the toolkit is offered as part of a comprehensive service for relatives.

## Competing interests

The authors declare that they have no competing interests.

## Authors' contributions

FL leads the design of the study, design of the intervention management of data collection and drafted the paper. DG co-wrote the intervention, facilitates recruitment, and supervises the clinical delivery of the support. LW contributed to design of the intervention, recruits and assesses participants, and manages the process of randomisation; VP contributed to the design of the intervention and management of the project; LC co-authored the intervention, co-manages the project WL co-authored the intervention and facilitates recruitment; GD provided statistical expertise to the protocol; GH co-authored the intervention and supports the management of the project; AP is instrumental in the recruitment and assessment of all participants including strategy development. All authors have contributed to the writing of this paper and have read and approved the final paper.

## Pre-publication history

The pre-publication history for this paper can be accessed here:

http://www.biomedcentral.com/1471-244X/11/100/prepub
